# Draft Genome Sequences of Marine Flavobacterium *Algibacter lectus* Strains SS8 and NR4

**DOI:** 10.1128/genomeA.01168-14

**Published:** 2014-11-13

**Authors:** Naoki Takatani, Masato Nakanishi, Pedro Meirelles, Sayaka Mino, Wataru Suda, Kenshiro Oshima, Masahira Hattori, Moriya Ohkuma, Masashi Hosokawa, Kazuo Miyashita, Fabiano L. Thompson, Ako Niwa, Toko Sawabe, Tomoo Sawabe

**Affiliations:** a Faculty of Fisheries Sciences, Hokkaido University, Hakodate, Japan; b Laboratory of Microbiology, Institute of Biology and Solid Action on Globalization and Environment, COPPE, Federal University of Rio de Janeiro (UFRJ), Rio de Janeiro, Brazil; c Center for Omics and Bioinformatics, Graduate School of Frontier Sciences, The University of Tokyo, Kashiwa, Japan; d Microbe Division/Japan Collection of Microorganisms, RIKEN BioResource Center, Ibaraki, Japan; e Department of Food and Nutrition, Hakodate Junior College, Hakodate, Japan

## Abstract

Here, we present the draft genome sequences of a zeaxanthin-producing flavobacterium, *Algibacter lectus* strains SS8 and NR4, isolated from coastal sediment and rock surfaces in Hakodate, Japan, respectively. This genomic information represents the first *Algibacter* genome sequences, which will help us to elucidate the biology and evolution of *Flavobacteriaceae* bacteria.

## GENOME ANNOUNCEMENT

*Flavobacteriaceae* is a group of bacteria containing a diverse array of Gram-negative, aerobic, nonmotile, pigmented bacteria that show yellow to orange colonies (1) and includes >114 genera (see http://www.bacterio.net/). One genus, *Algibacter*, was proposed in 2004 for gliding, orange-pigmented, facultatively anaerobic heterotrophs and currently consists of 10 species (2–4). *Algibacter lectus* is the type species of the genus *Algibacter* and in the proposal of the genus and species that produce orange nondiffusible pigments (2). We recently isolated the yellow agarolytic *A. lectus* strains SS8 and NR4. Strain SS8 produced zeaxanthin, which occupied up to 97% of the total carotenoids. However, as the genome structures of the strains have never been reported until now, the genomic plasticities of the *A. lectus* strains are still presently unknown.

The genome sequences of *A. lectus* strains SS8 and NR4 were sequenced with the Ion PGM system (Life Technologies, Carlsbad, CA). The genome sequences were *de novo* assembled using Newbler version 2.8. The annotation and genome analysis were performed by Rapid Annotations using Subsystems Technology (RAST) (5). The sizes of the draft genome of *A. lectus* strains SS8 and NR4 are 4,692,319 bp and 4,815,979 bp, comprising 56 and 72 contigs, respectively. Both have G+C contents of 33.3%. The redundancies are 18 and 17, and the *N*_50_ contig lengths are 194,285 bp and 205,911 bp, respectively. The numbers of putative coding sequences (CDSs) are 4,609 and 5,640, the numbers of rRNA sequences are 3 and 2, and the numbers of tRNA sequences are 38 and 37, respectively. Both strains possess a carotenoid biosynthesis gene cluster (encoding phytoene dehydrogenase, phytoene synthase, and β-carotene hydroxylase), as is the case with other *Flavobacteriaceae* bacteria. In addition, the lycopene cyclase was located downstream of the gene cluster. These genes are essential for zeaxanthin production (6). The orange-pigmented colony was reported in the *A. lectus* type strain (2). However, both *A. lectus* strains in this study formed yellow colonies. It is interesting to compare the genome to phenotype plasticity in carotenoid synthesis. This genome report will help reveal this difference. Strains SS8 and NR4 have been deposited in the Japan Collection of Microorganisms as JCM 19300 and JCM 19274.

### Nucleotide sequence accession numbers.

The genome data have been deposited in DDBJ/EMBL/GenBank under the accession numbers BBNQ01000001 to BBNQ01000056 and BBNU01000001 to BBNU01000072 for *A. lectus* strains SS8 and NR4, respectively.
